# Syphilinum in Cancerous Ulceration

**Published:** 1896-05

**Authors:** H. C. Morrow

**Affiliations:** Austin, Texas


### SYPHILINUM IN CANCEROUS ULCERATION.

H. C. Morrow, Austin, Texas.

In May, 1894, Dr. B., an allopathic physician, age, seventy years, consulted me. Has had for seven or eight years a number of sores on his face, which have been pronounced by several allopathic dermatologists to be cancerous in their nature. They came first as raw places on the face, and then covered with perfectly black scabs, which either do not come off or, if they do, leave raw sore places, which will not heal, but become again covered with the black scabs. Under each eye, and especially at the outer canthus of the right eye, the spots or sores look decidedly like epithelioma. The one under the corner of the right eye is threatening to involve the lower lid and the internal structure of the eye. On this eye, a few years since, there was an ulcer on the cornea, which nearly destroyed the sight. He can only distinguish daylight from darkness.

The conjunctiva of this eye is very red and inflamed, and there is ectropion of the lower lid. He is in bad health, and drinks a good deal of whisky. He had been treated by himself and all the "eminent" dermatologists and general practitioners in this part of the country and in New Orleans, and they had given the comforting assurance "that he might live several years, but that it would finally kill him." I put him on Syphilinum$^{cm}$ (Swan). To make a long story short, he has gradually improved with occasional relapses, until to-day he appears to be entirely well. He says for the first time in ten years there are no sores or scabs on his face. Where the worst ones were there are now cicatrices, but they look perfectly healthy and are gradually becoming smaller. The inflammation is entirely gone from his right eye, the ectropion is nearly removed, and he can see small objects six feet distant with his lame eye.

What perhaps is the most remarkable of all, he has stopped drinking whisky, thus verifying Dr. Thomas Wilde's observation, made some years since, upon the great efficacy of Syphilinum in alcoholism, although I am not prepared to agree with his second observation, that all chronic drunkards are syphilitics. My patient's skin troubles were always worse from the light and heat of the sun. There was but little local discomfort, but from the inroads the sore spot at the corner of his right eye was making on that structure, I have no doubt it was epithelioma.

There can be no question of the great value of this remedy for cancer. I believe every case of cancer to be the direct offspring of either syphilis or sycosis, or perhaps both, either acquired or inherited, and no case should be allowed to die or pass under the surgeon's knife, which alternative is about equivalent to death, without being given the benefit of a trial of Syphilinum.

